# Targeting Tumor-Associated Antigen: A Promising CAR-T Therapeutic Strategy for Glioblastoma Treatment

**DOI:** 10.3389/fphar.2021.661606

**Published:** 2021-06-24

**Authors:** Guidong Zhu, Qing Zhang, Junwen Zhang, Fusheng Liu

**Affiliations:** ^1^ Brain Tumor Research Center, Beijing Neurosurgical Institute, Capital Medical University, Beijing, China; ^2^ Department of Neurosurgery, Beijing Tiantan Hospital Affiliated to Capital Medical University, Beijing, China; ^3^ Beijing Laboratory of Biomedical Materials, Beijing, China; ^4^ Shandong Second Provincial General Hospital, Shandong Provincial ENT Hospital, Jinan, China

**Keywords:** car-t, glioblastoma, tumor-associated antigen, targeted therapy, immunotherapy, tumor microenvironment

## Abstract

Chimeric antigen receptor T cells (CAR-T) therapy is a prospective therapeutic strategy for blood cancers tumor, especially leukemia, but it is not effective for solid tumors. Glioblastoma (GBM) is a highly immunosuppressive and deadly malignant tumor with poor responses to immunotherapies. Although CAR-T therapeutic strategies were used for glioma in preclinical trials, the current proliferation activity of CAR-T is not sufficient, and malignant glioma usually recruit immunosuppressive cells to form a tumor microenvironment that hinders CAR-T infiltration, depletes CAR-T, and impairs their efficacy. Moreover, specific environments such as hypoxia and nutritional deficiency can hinder the killing effect of CAR-T, limiting their therapeutic effect. The normal brain lack lymphocytes, but CAR-T usually can recognize specific antigens and regulate the tumor immune microenvironment to increase and decrease pro- and anti-inflammatory factors, respectively. This increases the number of T cells and ultimately enhances anti-tumor effects. CAR-T therapy has become an indispensable modality for glioma due to the specific tumor-associated antigens (TAAs). This review describes the characteristics of CAR-T specific antigen recognition and changing tumor immune microenvironment, as well as ongoing research into CAR-T therapy targeting TAAs in GBM and their potential clinical application.

## Introduction

Glioblastoma is the most common and highly vascular malignant brain tumor that accounts for more than 50% of diagnosed intracranial glioma; it carries a poor prognosis, with a mean survival of ∼15 months (Zhang and Liu, 2020). The current first-line GBM treatments include surgery in combination with radiotherapy (RT) and temozolomide (TMZ) (Zhang Q. et al., 2018; Chen and Hambardzumyan, 2018). The dismal prognosis of GBM patients is due to the extreme proliferation, malignant invasion, immunosuppressive nature, intra- and intertumoral heterogeneity, and therapeutic resistance of GBM, as well as our limited understanding of the disease’s molecular characteristics and delayed diagnoses (Cheng et al., 2020; Zhang and Liu, 2020). It is therefore important to clarify the mechanisms of GBM pathogenesis and identify dependable and applicable molecular biomarkers.

GBM is widely recognized as an intractable disease. However, recent genomic studies have revealed that it undergoes continual mutation (Chen and Hambardzumyan, 2018). In view of gene expression differences, GBMs are further divided into three subgroups: proneural (PN), classical (CL), and mesenchymal (MES) (Chen and Hambardzumyan, 2018). The PN subclass is subdivided into two distinct types. The first is characterized by up-regulation of platelet-derived growth factor receptor A (PDGFRα) and deletion of the p53 gene, and the second has repeated mutations in the genes that encode isocitrate dehydrogenase (IDH1 and IDH2) (Boussiotis and Charest, 2018). The CL subtype has abnormal expression of wild-type or mutant epidermal growth factor receptor (EGFR) that is related to homozygous deficiency or mutation of the INK4a/ARF (CDKN2A) site and loss of phosphatase and tensin homolog (PTEN) function (Brennan et al., 2013). Finally, the MES subtype features loss of the neurofibromatosis type1 (NF1) gene function, and the driving nature of this change in GBM has been verified in NF1-deficient mouse models (Zhu et al., 2005). Understanding the molecular landscapes of GBM microenvironments can provide promising evidence and insight into specific gene treatments.

The GBM microenvironment consists of extremely intricate factors that lead to therapeutic resistance and disease progression (Zhang and Liu, 2020). Despite our improved understanding of the molecular characteristics of GBM, treatments targeting specific molecular pathways remain limited (Boussiotis and Charest, 2018). In recent years, tumor-associated antigens (TAAs) have gradually become a GBM research hotspot. TAAs can be over-expressed or down-regulated and regulate many features of GBM such as proliferation, migration, vascularization, immune evasion, and therapeutic limitation. For example, hepatocyte growth factor (HGF)/mesenchymal-epithelial transition factor (MET) expression results in enhanced proliferation, migration, and invasion due to phosphoinositide 3-kinase (PI3K)/protein kinase B (Akt) and focal adhesion kinase (FAK)/signal transducer and activator of transcription 3 (STAT3) signaling, while glioma cell invasion and increased angiogenesis share mechanisms of extracellular matrix (ECM) degradation through up-regulation of ECM-degrading proteases and activation of aberrant signaling (De Bacco et al., 2012; Joo et al., 2012; Cruickshanks et al., 2017; Keller and Schmidt, 2017). Moreover, programmed death ligand 1 (PD-L1) expression in the tumor microenvironment (TME) can induce T cell dysfunction and apoptosis by binding to programmed death protein 1 (PD-1), which works to promote tumor immune evasion (Sharpe and Pauken, 2018; Zamarin et al., 2018). Notably, tumor-specific receptors are only expressed on tumor cell surfaces, not in normal tissues (Liu et al., 2004). TAAs can produce ligand-independent component activity signals that enhance proliferation, accelerate tumor formation, decrease apoptosis, and promote GBM vascularization and invasion (Friese et al., 2004; Keller and Schmidt, 2017; Cheng and Guo, 2019).

Despite progress in understanding the molecular underpinnings and developing new treatments, the therapeutic outcomes are still poor; the life expectancy of patients with GBM is just 15 months (Zhang and Liu, 2020). The therapeutic modalities targeting TAAs are considerable and have yielded promising results in preclinical and clinical trials. A growing body of research shows that targeted therapies offer significant benefits. Moreover, CAR-T therapy has been approved for clinical trials for several solid tumors and showed good therapeutic safety and efficacy (O'Rourke et al., 2017; Tchou et al., 2017; Zhang et al., 2017). Currently, CAR-T therapy is of extreme interest for glioma treatment because it can specifically recognize and kill tumor cells and generate an anti-tumor immune microenvironment characterized by increased pro-inflammatory factors and decreased anti-inflammatory factors (Jin et al., 2018; O'Rourke et al., 2017). This review summarizes the biological characteristics and current research of TAAs in GBM, as well as their potential clinical application.

## Tumor-Associated Antigens in GBM

TAAs have been widely used as biomarkers for a variety of cancers and show differential expression in several GBM subtypes. Antigen expression can vary depending on the origin, stage, and grade of tumor, and even the examination method (Boussiotis and Charest, 2018). Many kinds of molecules have been found in GBM, including tumor-suppressing and -promoting proteins. Increasing evidence suggests that GBM-associated antigens with various functional proteins are abundant and play crucial roles in glioma initiation, progression, and recurrence (De Bacco et al., 2012; Liu et al., 2015; Avril et al., 2017). This review summarizes the biological characteristics of antigens in GBM.

## EGFR/EGFRvIII

**FIGURE 1:**
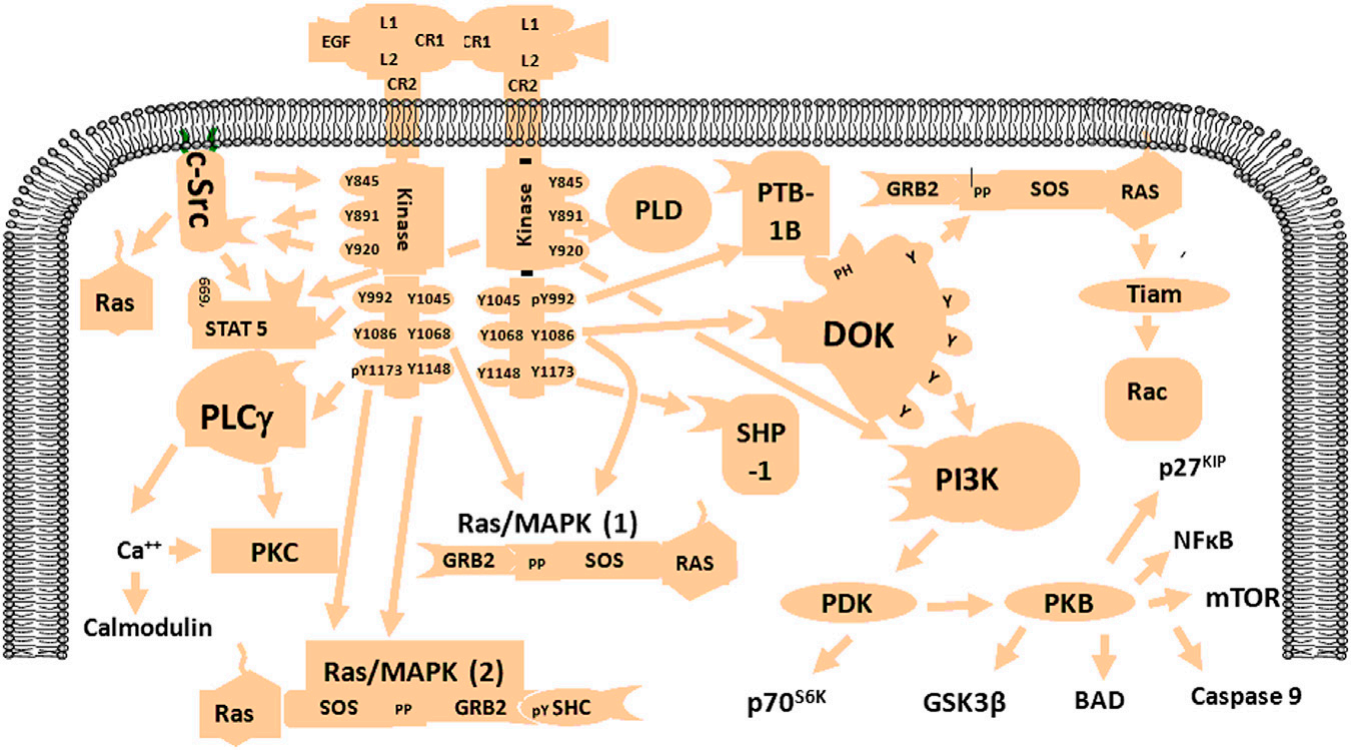
Schematic diagram of the EGFR signaling network. EGFR is activated by specific factors that induce different signaling pathways such as PI3K/Akt, Ras/MAPK, NF-kB, and mTOR with various biological functions including enhancement of proliferation, invasion, angiogenesis, and resistance to apoptosis.

Epidermal growth factor receptor (EGFR) is a tyrosine kinase receptor binds EGF, which belongs to the ErbB family, so it is also called human epidermal growth factor receptor-1 (HER1) or ErbB1. This family contains four receptors (ErbB1-4 or HER1-4), but EGFR is the best characterized (An et al., 2018). Genomic analyses revealed that more than half of the EGFR genes are changed in rat GBM due to amplifications and mutations (Eskilsson et al., 2018). EGFR gene alterations include point mutations or deletions that can lead to constitutive activation of the receptor independently of the ligand (Liu et al., 2015; An et al., 2018; Eskilsson et al., 2018). The most common deletion is exons 2–7 of the EGFR extracellular domain, leading to truncated mutant III (EGFRVIII) (Furnari et al., 2015). The cellular processes activated by EGFR or receptor mutations may depend on specific cell types (Liu et al., 2015). Activated EGFR may be involved in multiple signaling pathways, (e.g. PI3K/Akt, Ras/Raf/MEK/ERK, STAT3, and phospholipase Cγ [PLC]) with various functional roles including proliferation, invasiveness, vascularization, and apoptotic resistance (Liu et al., 2015; An et al., 2018; Eskilsson et al., 2018). The EGFR signaling pathway is shown in Figure 1.

EGFRVIII mutants are found in GBM but not in normal brain tissue (Yang et al., 2017). The kinase activity of EGFRVIII is essential for enhancing the signaling pathway. EGFRVIII signals differ from those of wild-type EGFR in both quantity and quality. EGFRVIII has a low level of constitutive kinase activity due in part to impaired endocytosis and degradation (An et al., 2018). EGFRVIII can transduce signals through the traditional EGFR pathway and is regulated by RAS/mitogen-activated protein kinase (MAPK), PI3K/Akt, and JAK/Stat. EGFR/EGFRVIII may take part in the genesis and development of glioma, including proliferation, migration, tube formation, and drug resistance (Liu et al., 2015; Eskilsson et al., 2016; Keller and Schmidt, 2017). Therefore, EGFR/EGFRVIII could serve as diagnostic and prognostic biomarkers for patients with GBM.

## PDGFRA

PDGFRA is a transmembrane receptor containing five immunoglobulin-like domains and one tyrosine kinase domain. Ligand-receptor binding activates key downstream signal transduction pathways that promote tumorigenesis, including MAPK, PI3K/Akt, JAK/Stat, and PLC-protein kinase C (Alentorn et al., 2012). Although PDGFRA amplification is not as common as EGFR amplification, it is found in 11% of GBMs, making it the second most common tyrosine kinase receptor gene amplification in this tumor family (Ozawa et al., 2010; Alentorn et al., 2012). In 2013, Phillips et al. showed that PDGFRA amplification increased with grade and was associated with poor prognosis in IDH1-mutant GBMs (Phillips et al., 2013). Amplification or activation mutations in PDGFRA represent a significant subset of PN GBM, and up-regulation of PDGFRA promotes GBM initiation and progression (Silber et al., 2012). Previous work showed that the PDGFRA gene is rearranged in GBM (Ozawa et al., 2010). PDGFRA gene expression is mediated by various mechanisms in patients with high-grade glioma, and its amplification may be a prognostic biomarker.

## IL13Rα2

Interleukin-13Rα2 (IL-13 receptor α2) is a monomeric high-affinity receptor that is up-regulated by more than 50% in GBM; it is a prognostic marker of poor survival in patients but is not significantly expressed in normal brain tissue (Brown et al., 2013; Thaci et al., 2014). In high-grade gliomas, IL13RA2 expression was closely correlated with the MES GBM, which may reflect its association with the pro-inflammatory features of this subtype (Brown et al., 2015). IL13Ra2 plays a crucial role in GBM invasiveness and progression. Moreover, IL-13Rα2 is an inhibitor of IL-4-dependent signal transduction and gene expression in the STAT6 response. This restriction may be controlled by an interaction between the short intracellular domain of the IL-13Rα2 protein and the cytoplasmic domain of the IL-4Rα chain (Rahaman et al., 2002). Early clinical research supports the safety and tolerability of vaccine therapies targeting IL13Rα2 and IL-13 immunotoxin for GBM (Brown et al., 2015).

## NKG2D

Natural Killer Group 2 member D (NKG2D) is a C-type lectin-like homodimeric receptor expressed on human NK, ϒδT, and CD8^+^ αβT cells (Friese et al., 2004). The NKG2D ligand (NKG2DL) is a key element of tumor immune surveillance. NKG2DL can trigger self-ligands, and such markers mediate the killing effect of NK and CD8^+^ T cells. In 2018, Fluh et al. demonstrated that NKG2DL was positively expressed in glioma stem cells (GSCs) *in vivo* and *in vitro* (Fluh et al., 2018). The over-expression of NKG2D in CD8^+^ T and NK cells could enhance the immune response to hinder glioma cell migration and invasion and eradicate tumors (Friese et al., 2004). NKG2D also meditates the innate reactivity of Vγ9Vδ2 T lymphocytes and targets GBM cells by recognizing pathways involved in the innate identification of the MES subtype (Chauvin et al., 2019). These results underscore the utility of novel targeted immunotherapies in GBM treatment.

## HER2

**FIGURE 2:**
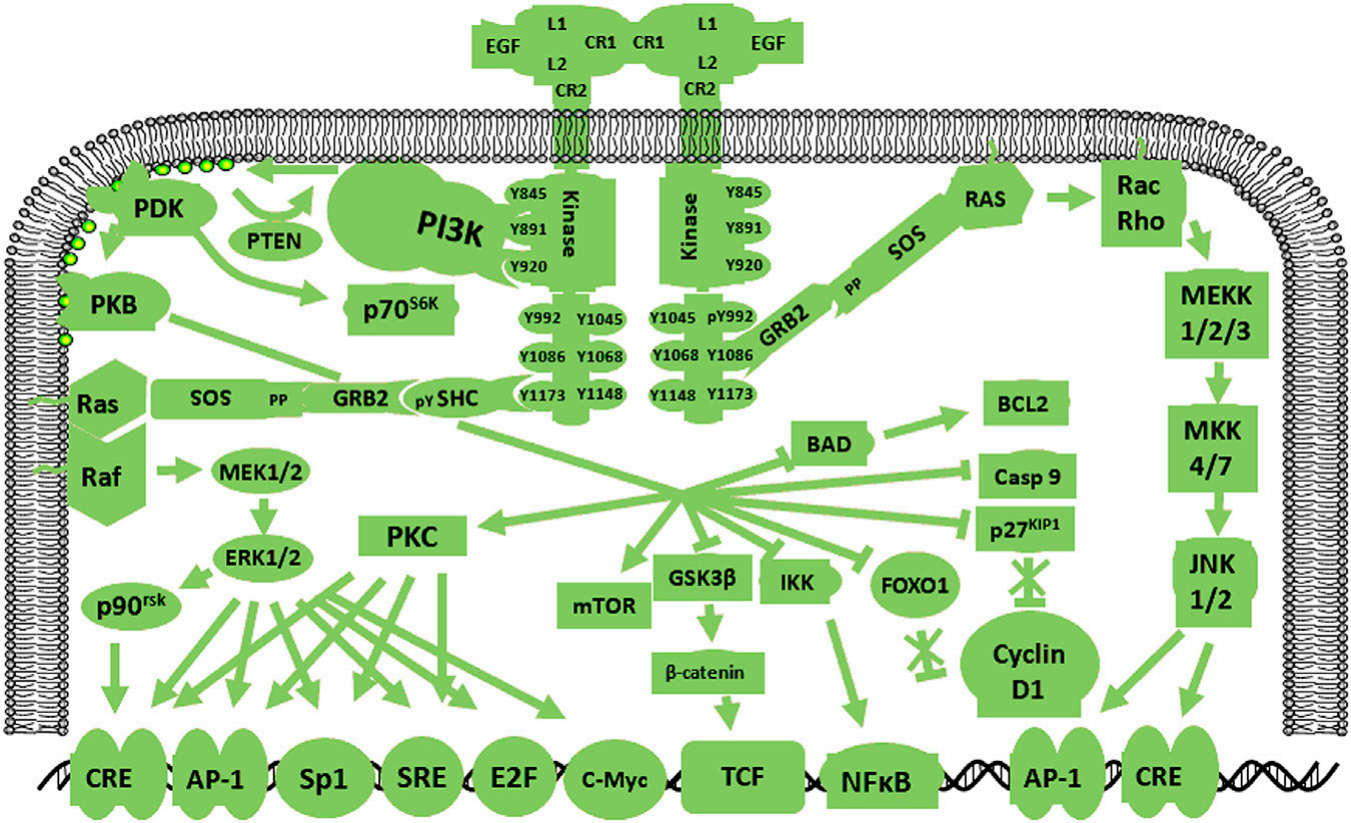
Schematic diagram of the HER2 signaling pathway. HER2 amplification in human GBM is limited by the *EGFR* gene, indicating that HER2 expression in GBM is closely related to the EGFR. HER2 pathway activation gives rise to cascades of specific signals.

Human epidermal growth factor receptor 2 (HER2), also known as HER2/neu or ErbB2, is a 185-kDa transmembrane protein that is broadly homologous to the EGFR in structure and sequence. HER2 gene magnification and up-regulation frequently occur in many human malignancies (Koka et al., 2003; Liu et al., 2004). HER2 expression in GBM cells and the identification of peptide-specific, major histocompatibility complex-limited CTL production in GBM cells lay the foundation for the use of relevant alternative assays to measure antigen-specific cytotoxicity (Liu et al., 2004). Moreover, ErbB gene amplification is limited to the EGFR gene in human GBM, indicating that ErbB2 expression in GBM is closely associated with EGFR levels (Schlegel et al., 1994). HER2 signaling pathways are shown in Figure 2. Previous work showed a relationship between HER2/neu expression in GBM and survival time. Specifically, HER2/neu over-expression in the early stage of GBM is used to predicting mortality (Koka et al., 2003). These results indicate that HER2 may be a poor prognostic indicator for patients with GBM.

## MET

**FIGURE 3:**
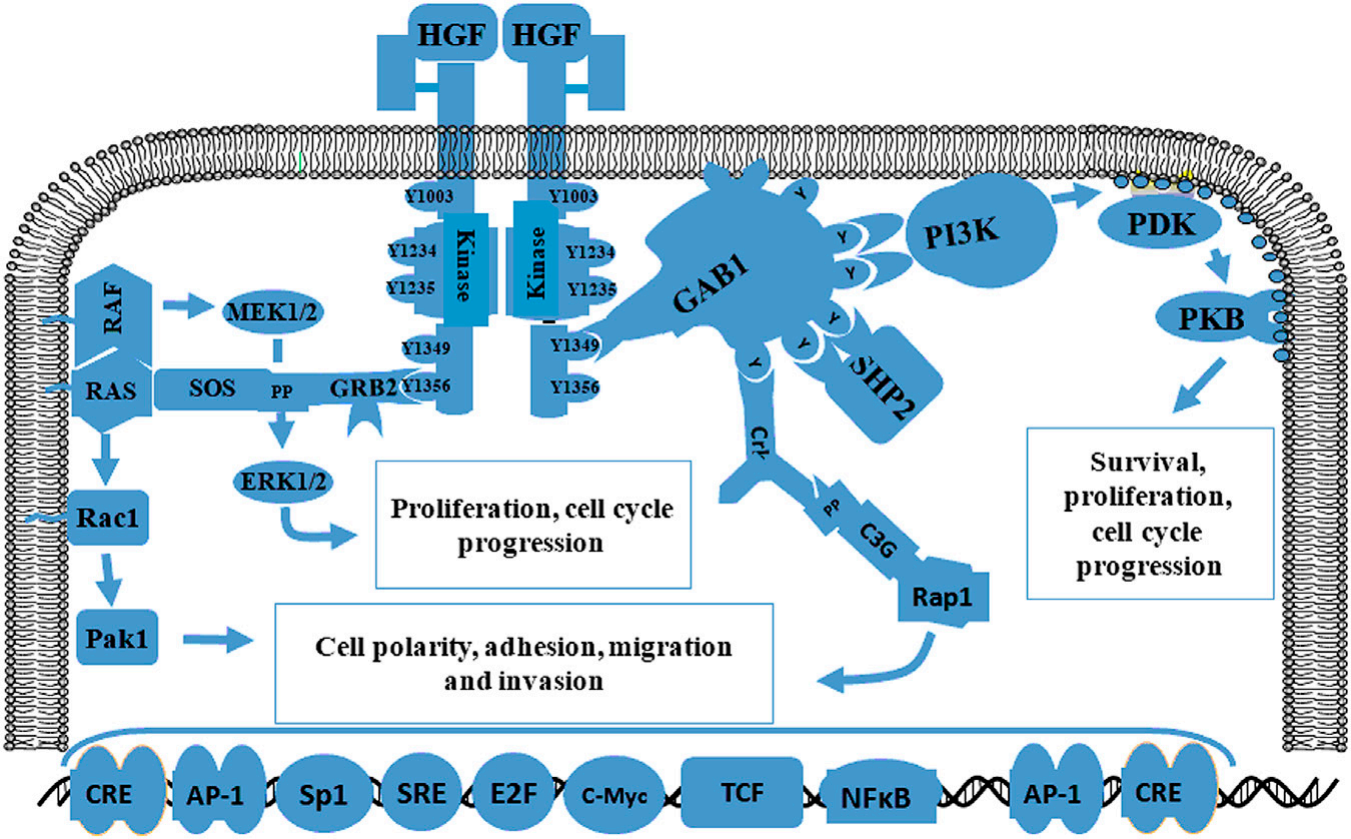
Schematic diagram of the MET signaling pathway related to proliferation, migration, and invasion. The initiation of MET signaling activates several signals including Gab1, Grb2, Ras, SOS and Shp2 and the subsequent phosphorylation of downstream molecules such as STAT3 and Ras/MAPK/ERK. The interaction between MET and HGF plays a crucial role in proliferation, survival, migration, invasion, treatment resistance, and GBM recurrence.

MET and HGF are located on chromosome 7q31 and 7q21.1, respectively (Cruickshanks et al., 2017). The HGF receptor MET is a high-affinity tyrosine kinase composed of α and β subunits. The extracellular domain consists of an α-subunit and amino-terminal region of the β-subunit. The other β-chain regions span the cell membrane to create a cytoplasmic region with tyrosine kinase activity (Cheng and Guo, 2019). In 2012, Joo et al. demonstrated that MET signaling plays vital roles in the maintenance, migration, and radiation resistance of GSCs (Joo et al., 2012). The mutual effect of MET and HGF induces auto-phosphorylation at various tyrosine remainders, which generates stimulation of a few signaling molecules such as Gab1, Grb2, Src, Shc, PLC-γ, and c-Cbl that are subsequently phosphorylated to induce downstream transduction including STAT3 and Ras/MAPK/ERK, (De Bacco et al., 2012; Joo et al., 2012; Cruickshanks et al., 2017; Cheng and Guo, 2019). MET and HGF signaling pathways are shown in Figure 3. Some studies confirmed that the interaction between MET and its ligand HGF plays an important role in GBM proliferation, invasion, survival, vascularization, treatment resistance, and recurrence (Hu H. et al., 2018; Cheng and Guo, 2019). In addition, glioma expressing PTPRZ1-MET (ZM) gene fusions show potent invasiveness and sensitivity to chemotherapy drugs (International Cancer Genome Consortium PedBrain Tumor Project, 2016; Hu H. et al., 2018). In summary, MET is a functional marker and a candidate target for novel GBM treatments.

## Immune Checkpoint Proteins

GBM is a highly immunosuppressive malignant brain tumor. Immune checkpoint proteins—especially PD-1, PD-L1, cytotoxic T lymphocyte-associated protein 4 (CTLA-4), and B7 homolog 3 (B7-H3) —have immunosuppressive characteristics that are up-regulated in the GBM TME, which contribute to tumor cell immune escape (Berghoff et al., 2015; Nduom et al., 2016; Saha et al., 2017; Wang et al., 2018). CTLA-4 is an immune checkpoint co-inhibitory receptor expressed on T cells that competes with the co-stimulatory receptor CD28 for binding by their ligands CD80 and CD86; this reduces the number of T helper cells and effector T cells while increasing regulatory T cells (Tregs) (Liu F. et al., 2020). PD-1 is another immune inhibitor expressed on T cells, B cells, and NK cells. Its ligand PD-L1 is up-regulated in GBM cells. PD-L1 binds PD-1 and induces immune cell dysfunction (Litak et al., 2019). The well-studied immune checkpoints including CTLA-4, PD-1, and PD-L1 play crucial roles in the tumor immunoreaction process (Fong et al., 2012; Wang et al., 2019). Moreover, higher expression of immune checkpoint proteins is related to poor prognosis in patients with GBM. Various clinical studies targeting PD-1/PD-L1 and CTLA-4 have been carried out in patients with GBM to promote powerful antitumor immune responses (Fong et al., 2012; Litak et al., 2019; Liu F. et al., 2020).

B7-H3 is another immune checkpoint protein that belongs to the immunoglobulin superfamily and is considered to be involved in mediating the T cell immune response (Picarda et al., 2016; Wang et al., 2018). Previous research described its biological characteristics in malignant brain gliomas and found that its expression was closely correlated with cell cycle, immune response, and angiogenesis in glioma. Moreover, B7-H3 levels were higher in high-grade compared to low-grade tumor tissues. Elevated B7-H3 expression is associated with extremely poor prognosis. It is up-regulated in wild-type IDH gliomas and the MES subtype (Zhang C. et al., 2018). In 2020, Zhong et al. demonstrated that B7-H3 contributed to epithelial-mesenchymal transition (EMT) development via E-cadherin down-regulation and MMP2/9 up-regulation and promoted glioma progression and invasion through JAK2/STAT3/Slug-dependent signaling (Zhong et al., 2020). Moreover, the collaboration of B7-H3 with other checkpoint members may result in T cell dysfunction. Further research into B7-H3 will provide promising insights to further optimize GBM immunotherapies.

## Tumor Suppressor Proteins

It is well known that tumor suppressor genes play vital roles in tumorigenesis. Here we mainly summarize PTEN and P53 because mutations in these genes contribute to tumorigenesis. Previous studies showed that PTEN and P53 mutations occurred in GBM patients with extremely poor prognosis (Wiencke et al., 2007; Benitez et al., 2017; Ham et al., 2019). PTEN is a candidate gene for inactivating mutations in GBM. Deletions and mutations in PTEN in GBM are frequent events that are related to treatment resistance. Benitez et al. found that PTEN could regulate tumorigenesis through chromatin-associated complexes of death-domain associated protein and histone H3.3 (Benitez et al., 2017). P53 is another tumor suppressor that participates in different aspects of cell cycle regulation and conversion inhibition (Ham et al., 2019). TP53 mutations are related to poor prognosis and EMT characteristics in malignant gliomas (Cho et al., 2017).

## CAR-T Research Advances in GBM

### Targeting TAAs

**FIGURE 4:**
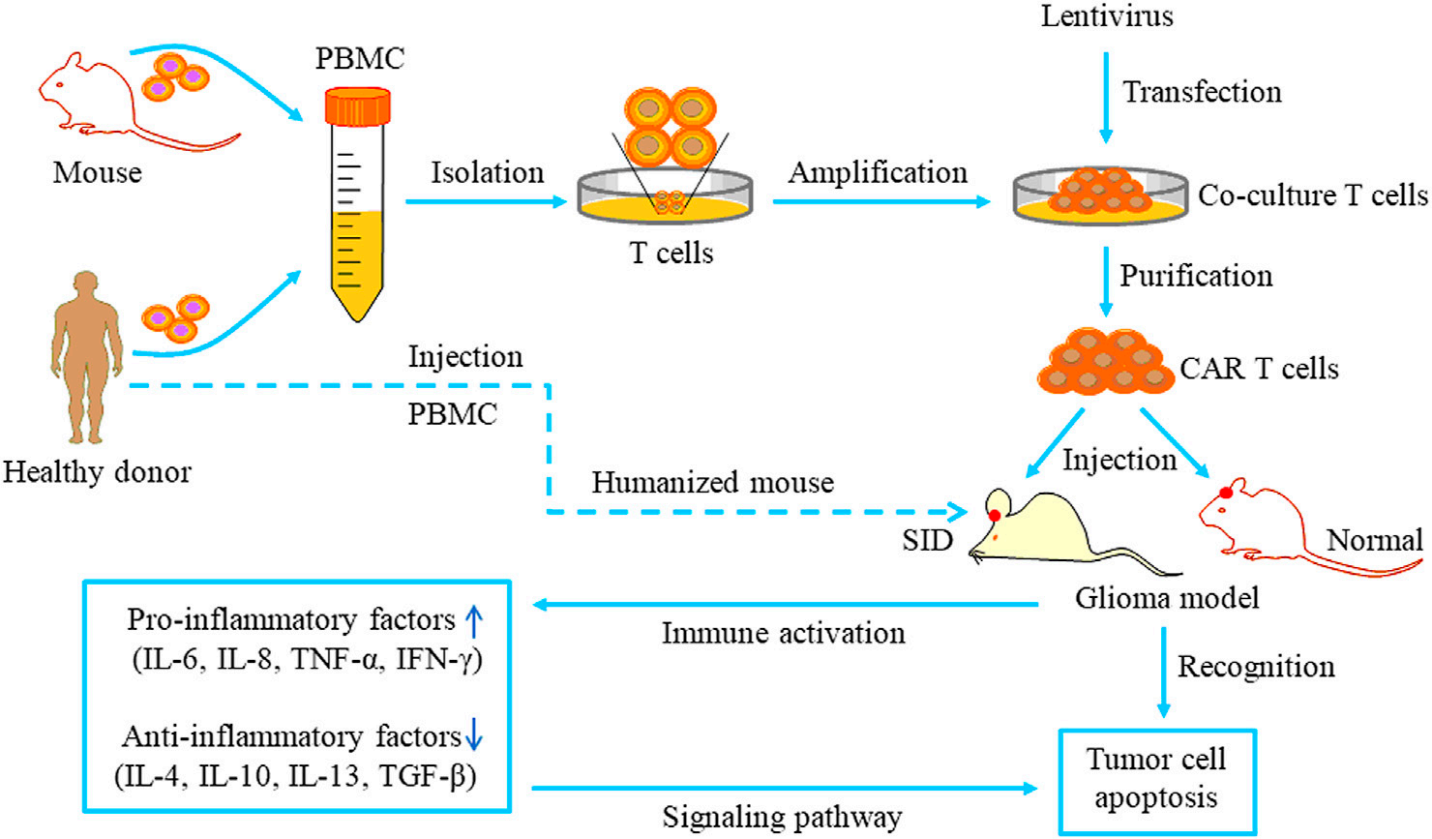
The pattern of CAR T cell-based therapy studies for glioma. T cells are separated from the peripheral blood of healthy donors or mice and cultured in special media. The lentivirus carrying a specific ligand is transduced into the cultured T cells. Then the purified CAR T cells are injected into orthotopic glioma models and recognize tumor cells via specific binding to antigen receptors. Immune system activation can increase pro-inflammatory events and reduce anti-inflammatory events, ultimately inducing tumor cell apoptosis via specific signaling pathways.

GBM is a highly intractable disease. Despite great progress in molecular research and treatments, the clinical outcomes remained extremely poor, and the survival of patients with GBM has not been significantly improved. Developing targeted GBM therapies has increasingly become a research hotspot. Several preclinical and clinical studies on CAR-T immunotherapy for malignant gliomas have yielded positive results (Ahmed et al., 2017; Schneider et al., 2017; Thistlethwaite et al., 2017; Haydar et al., 2020). The therapeutic patterns of CAR-T cells targeting TAAs for GBM treatment are depicted in Figure 4 **.** CAR-T therapy can specifically recognize and kill tumors and create an anti-tumor immune microenvironment. These targeted therapies will be gradually developed to provide therapeutic approaches for gliomas.

### Targeting EGFRvIII

Exploration of glioma-specific antigens identified many CAR-T strategies for glioma. A clinical study confirmed that CAR-T targeting EGFRvIII can specifically kill GBM cells by releasing cytokines and induced glioma cell toxicity in a dose-and time-dependent manner for patients with recurrent GBM (O'Rourke et al., 2017). Another group showed a close correlation between CAR-T activity and the concentration of small molecules that increased the safety of CAR-T treatment (Zheng et al., 2018). Bispecific T-cell engager (BiTEs) have an anti-EGFR effect, and Choi et al. found that EGFR-targeted BiTEs generated by CAR-T can generate powerful and specific antitumor activity that mitigates the effect of EGFRVIII antigen loss (Choi et al., 2019). The combination of EGFRvIII-CAR-T and PD-1 down-regulation has potent anti-glioma efficacy and prolongs survival in mice. Furthermore, PD-1 knockout significantly enhances lysis of CAR-T cells targeting EGFRvIII for PD-L1^+^ EGFRvIII^+^ GBM cells (Song et al., 2020; Zhu et al., 2020a; Zhu et al., 2020b). Importantly, intratumoral IL-12 administration effectively reshapes the TME (i.e., increased pro-inflammatory CD4^+^T cells and decreased Tregs), thus improving CAR-T cell immunotherapy in a preclinical model (Agliardi et al., 2021). These results provide insight into developing new CAR-T strategies for GBM therapy in future.

### Targeting IL13Ra2

IL13Ra2-CAR-T is a promising immunotherapy strategy for GBM. In 2015, a clinical trial was performed to evaluate effectiveness and safety of IL13Rα2-redirected CAR-CD8^+^ T cells in patients with recurrent GBM (Brown et al., 2015). This clinical experience in the intracranial treatment of GBM with IL13Rα2-specific CAR-T lays the foundation for the future application of modified CAR-T therapies. This approach creates a pro-inflammatory immune microenvironment in a syngeneic model of GBM by generating obvious increases in CD4^+^ and CD8^+^ T cells, while decreasing the number of myeloid-derived suppressor cells and producing cytokines such as interferon (IFN)-γ and tumor necrosis factor-a, thus further enhancing anti-glioma effects (Pituch et al., 2018). Brown and colleagues designed a 4-1BB (CD137) co-stimulatory CAR (IL13BBѯ) and a manufacturing platform using enriched central memory T cells to confirm that IL13Ra2-targeted CAR-T enhanced anti-tumor effect sin patient-derived tumor models (Brown et al., 2016; Brown et al., 2018). In a clinical trial, CAR-T cells targeting IL13Rα2 had potent antitumor effects in patients with malignant glioma (Brown et al., 2016). In addition, human IL13Rα2-CAR-T therapy improves the GBM immune microenvironment and induces the activation of host immune cells that enhance the antitumor efficacy of CAR-T (Alizadeh et al., 2021). These findings will contribute to the clinical application of CAR-T for malignant gliomas.

### Targeting NKG2D

A previous study demonstrated that chemotherapy and RT enhanced NKG2DL expression for all GBM models, and tissue samples from GBM patients show up-regulation of NKG2DL expression after treatment with TMZ or RT (Weiss et al., 2018a). This suggests improved glioma cell immunogenicity and provides a theoretical basis for combining NKG2D-based immunotherapy with TMZ and RT. In 2019, Yang et al. showed that NKG2D specific-CAR T effectively eradicated GBM cells and GSCs *in vitro* and induced cytokine over-expression (Yang et al., 2019). CAR-T significantly inhibited tumor growth *in vivo* and did not show obvious treatment toxicity in GBM-bearing animals. CAR-T targeting NKG2D combined with RT exerts synergistic efficacy in mouse glioma models (Weiss et al., 2018b). The findings described above provide a basis to develop rationale immunotherapeutic strategies to treat human glioma patients in the future.

### Targeting B7-H3

B7-H3 is a new target of CAR-T cells for GBM therapy. Tang et al. evaluated the antitumor activities of CAR-T targeting B7-H3 in both primary glioma cells and GBM cell lines; they also observed significantly longer median survival in the CAR-T group compared to the control group in an orthotropic GBM model (Tang et al., 2019). In 2019, Nehama et al. verified that CAR-T targeting B7-H3 release effective factors such as IFN-γ and IL-2 and control the growth of B7-H3^+^ human GBM cell lines and neurospheres (Nehama et al., 2019). CAR-T targeting B7-H3 has potent antitumor activities in patient-derived orthotopic xenograft and immune-competent animal models (Haydar et al., 2020). Dual CAR-T target antigens exhibit enhanced antitumor activities and improve the heterogeneity and variation of antigens in treating multiple types of solid tumors (Yang et al., 2020). These lines of evidence suggest that B7-H3 may be a promising target for CAR-T treatment for GBM.

## Potential Clinical Applications

### TAAs Are Promising Molecular Biomarkers

TAAs have been used as targets for glioma therapy in basic studies and clinical trials. These results hold great promise for patients with refractory disease. Several GBM-associated biological molecules have been extensively studied. An important indicator is how representative they are in the overall tumor or at least reflect the tumor’s major biological characteristics. Variable expression in GBM is one of the main causes of tumor heterogeneity, which contributes to treatment resistance and poor efficacy. A growing number of studies have confirmed a key role of TAAs in glioma initiation and development. Targeted GBM therapy is increasingly researched due to limitations of traditional therapeutic strategies. Hence, TAA-specific targeted therapies have a good prospect of clinical application in glioma treatment and are receiving more attention.

Molecular detection is widely used for clinically monitoring patients with brain malignant tumors. One study reported up-regulation of glioma-associated antigens and found that their expression correlated with histological grade and patient prognosis (Brown et al., 2013; Jin et al., 2018). Therefore, TAAs have potential as diagnostic and prognostic markers. Acquired drug resistance is an important issue that restricts treatment. Timely detection of potential drug-resistant patients who may require alternative treatment is very important to improve prognosis. Collectively, the existing evidence indicates that TAAs in GBM show great potential as biomarkers, and their clinical application is worthy of further investigation.

### TAAs in GBM Treatment

Considering the important roles of TAAs in glioma, therapies that target TAAs will be important treatment options. In 2018, Hu et al. reported that the MET kinase inhibitor PLB1001 demonstrated significant ability to selectively inhibit tumor cells with MET mutations in an animal model. Moreover, the drug was able to pass through the blood-brain barrier (BBB) and was used in a phase I clinical trial of patients with chemo-resistant glioma (Hu H. et al., 2018). We designed a novel Ki67-expressing oncolytic adenovirus to target glioma and showed potent oncolytic efficacy (Zhang Q. et al., 2020). These studies underscore the possibility of targeting specific antigens for GBM therapy.

Administering chemotherapy for GBM faces great challenges and limitations because of the BBB (Moesta et al., 2017). However, modified CAR-T have special features such as high proliferation, rapid onset, high remission rate, and long remission time that can overcome dominant biological barriers such as the BBB. In 2018, Samaha et al. modified CAR-T cells to successfully cross the BBB and spread tumor sites, which contributed to glioma eradication and significantly extended animal survival (Samaha et al., 2018). Therefore, CAR-T could be a promising glioma therapy delivery system.

Compared with T cells, CAR-NK (natural killer) cells have a few advantages such as their safety in clinical trials, their mechanism for recognizing tumor cells, and high levels in clinical specimens (Hu Y. et al., 2018). EGFR-CAR-NK cells induce potent cell lysis and increase IFN-γ production when co-cultured with GBM cells, and intracranial NK-EGFR-CAR cell injection can effectively inhibit tumor growth and significantly extend the life expectancy of orthotopic GBM animal models (Han et al., 2015). In addition, human CAR-NK cells expressing a HER2-specific show potent antitumor immunity in NSG GBM models (Schonfeld et al., 2015; Zhang et al., 2016). Many modalities have been exploited to increase the safety and efficacy of CAR-based immunotherapies. These given findings suggest TAAs-specific-CAR cells represent a prospective strategy for GBM treatment.

### Oncolytic Virus in Combination With CAR-T for GBM Treatment

In recent years, characterizing complex TMEs has become a research hotspot in tumor therapy (Gutmann, 2015; Wang et al., 2017; Xie et al., 2019). GBM is a highly immunosuppressive tumor, which results in immunotherapy resistance and limitation (Saha et al., 2017). Immunotherapy is an emerging therapeutic strategy for solid tumors. Although CAR-T therapy shows potential advantages compared to other immunotherapies, they cannot function effectively because of the immunosuppressive TME of GBM. The TME can be regulated by oncolytic viruses (OVs) that stimulate antitumor immunity (Kurozumi et al., 2007; De Silva et al., 2010; Moncayo et al., 2018; Meng et al., 2019; Liu et al., 2020b), but the potential mechanisms remain unclear. Researchers have exploited the advantages of OVs to improve CAR-T for solid tumor therapy. For example, mice treated with a combined injection of OVs and CAR-T cells show long-term antitumor immune protection.

OVS have therapeutic capacity for cancer treatment. During infections, viruses hijack host cells replication mechanisms, promote their own genetic expression, and unavoidably cause the death of host cells, allowing viral offspring to reach and infect neighboring host cells (Martinez-Velez et al., 2019). OVs can specifically infect and kill tumor cells and have become an attractive treatment choice (Fang et al., 2014; Martikainen and Essand, 2019; Zhang W. et al., 2020). Our research team has showed that OVs have potent therapeutic efficacy and safety to treat malignant brain glioma and uveal melanoma (Zhang J. et al., 2020; Liu et al., 2020a; Zhang Q. et al., 2020; Liu et al., 2020b; Liu et al., 2021). Previous studies confirm that intratumoral OV injection can enhance anti-tumor immunity and eliminate contralateral tumors (Lun et al., 2005; Saha et al., 2018; Martinez-Velez et al., 2019; Liu et al., 2021). Moreover, OV-mediated tumor tissue destruction is closely associated with innate immune responses and adaptive T-cell immune responses (Hellums et al., 2005; Stephenson et al., 2012). Immune checkpoint molecules such as PD-1 and CTLA-4 are often up-regulated in the TME and induce T cell dysfunction (Saha et al., 2017; Boussiotis and Charest, 2018). However, OVs direct immune cells into tumor regions, further regulating the TME and generating antitumor immune responses. OVs equipped with immunomodulatory factors deliver therapeutic genes and further induce immune cell activation and proliferation (Lei et al., 2009; Lun et al., 2010; Jiang et al., 2017). For example, IL-15 enhances the activities of macrophages and neutrophils, accelerates the production and activation of NK cells and CD8^+^ T cells, and regulates memory T cell survival and proliferation (Stephenson et al., 2012). A recent study revealed that combining CD19-CAR-T cells and OVs encoding for truncated CD19 (OV19t) achieved a >50% cure rate in a mouse tumor model by inducing local immunity characterized by tumor T cell infiltration; CAR T cell-mediated tumor killing also promoted viral release from dying tumor cells, further propagating CD19t tumor expression (Park et al., 2020). Combination treatment overcomes the limitation of targeting CAR T to solid tumors and helps normalize the TME, and these effects will improve CAR-T therapeutic efficacy in GBM.

## Conclusion

As new targeted therapies are developed, an increasing number of novel therapeutic strategies have achieved many beneficial outcomes, such as CAR-T immunotherapy that can specifically recognize and target tumor cells. In GBM treatment, second- and third-generation CAR-T therapies have shown promising therapeutic efficacy to prolong patient survival. However, there have been no phase III clinical trial results due to the challenging location and particular characteristics of GBM. Further studies are needed to optimally modify CAR-T cells and their targets to improve efficacy. The current dilemmas that must be addressed are selecting the best delivery approach, comprehending the TME, exploiting new targets, and improving therapeutic efficacy. As we learn more about these issues, we expect a bright future for CAR-T therapy for GBM.

## Abbreviations

CAR-T: chimeric antigen receptor T cells, CTLA-4: cytotoxic T lymphocyte-associated protein 4, GBM: glioblastoma, EGFR: epidermal growth factor receptor, EGFRvIII: epidermal growth factor receptor mutant variant III, HER2: human epidermal growth factor receptor 2, IL13Rα2: interleukin-13 receptor α2, MET: mesenchymal-epithelial transition factor, NKG2D: natural killer group 2 member D, OV: oncolytic virus, PBMC: peripheral blood mononuclear cell, PD-1: programmed death 1, PDGFRα: platelet-derived growth factor receptor A, PTEN: phosphatase and tensin homolog, TAA: tumor-associated antigen, TME: tumor microenvironment.
